# Plasma nesfatin-1 and DDP-4 levels in patients with coronary artery disease: Kozani study

**DOI:** 10.1186/s12933-021-01355-x

**Published:** 2021-08-13

**Authors:** Nikolaos P. E. Kadoglou, Emmanouil Korakas, Stylianos Lampropoulos, Eirini Maratou, George Kassimis, Nikolaos Patsourakos, Panagiotis Plotas, Paraskevi Moutsatsou, Vaia Lambadiari

**Affiliations:** 1 Medical School, University of Cyprus, 215/6 Old road Lefkosias-Lemesou, Aglantzia, 2029 Nicosia, Cyprus; 2 2nd Department of Internal Medicine, Research Institute and Diabetes Centre, Athens University Medical School, Attikon University General Hospital, Athens, Greece; 3 Cardiology Department, “Mamatsio” General Hospital of Kozani, Kozani, Greece; 4 2nd Cardiology Department, “Hippokration” Hospital, Aristotle University of Thessaloniki, Thessaloniki, Greece; 5 Cardiology Department, “Tzaneio” General Hospital of Piraeus, Pireas, Greece; 6 Department of Cardiology, University of Patras Medical School, Patras, Greece; 7 Department of Clinical Biochemistry, Medical School, National and Kapodistrian University of Athens, Athens, Greece

**Keywords:** Nesfatin-1, Dipeptidyl peptidase-4, Coronary artery disease, Unstable angina

## Abstract

**Background:**

Nesfatin-1, a novel adipokine and dipeptidyl peptidase-4 (DPP4), a mam malian serine protease, are potent factors of atherosclerosis. In the present cross-sectional study, we investigated whether the plasma nesfatin-1 and DPP4 is associated with the prevalence and severity of coronary artery disease (CAD) with and without diabetes mellitus (DM).

**Methods:**

We consecutively enrolled a total of 240 patients with significant CAD (previous revascularization or angiographically-proven coronary artery stenosis > 50%) presented with either unstable angina (UA, N = 76) or stable chronic CAD (SCAD, N = 165). 85 patients with at least 2 classical cardiovascular risk factors but without significant CAD served as controls. The severity of CAD was assessed using coronary angiography by the Gensini score. Clinical parameters, glycemic and lipid profile, high-sensitivity CRP (hsCRP), nesfatin-1 and DPP4 levels were assayed.

**Results:**

No differences were found for age, sex, hypertension and diabetes distribution between groups. Low nesfatin-1 levels were found in both CAD groups (UA & SCAD) with respect to controls. The difference between UA and SCAD groups was marginally non-significant. There was a significant increase of DPP4 along UA to SCAD and control groups. Differences between groups remained unchanged in non-diabetic participants. Nesfatin-1 significantly correlated to hsCRP (r = − 0.287, p = 0.036), HOMA-IR (r = − 0.587, p = 0.007) and hyperlipidemia (r = − 0.331, p = 0.034). DPP4 was significantly associated with hs-CRP (r = 0.353 p < 0.001) and FPG (r = 0.202, p = 0.020) in univariate analysis, but those correlations were lost in multiple regression analysis. There was a negative correlation between nesfatin-1 and the severity of CAD, quantified by the Gensini score (r = − 0.511, p < 0.001), but no association was found for DPP4.

**Conclusions:**

Serum DPP4 levels are increased in patients with CAD, while serum nesfatin-1 levels have a negative association with both the incidence and the severity of CAD. These results are independent of the presence of diabetes mellitus. In addition, both peptides have a strong association with hsCRP.

*Trial registration* ClinicalTrials.gov Identifier: NCT00306176

## Introduction

Nesfatin-1 is a relatively novel adipokine which was discovered by Oh et al. [1]. It is secreted from the hypothalamic nuclei and it was initially considered a satiety molecule which exerts anorexigenic effects through the melanocortin system, being decreased by fasting and increased on refeeding [2]. In addition to its role in metabolic regulation, nesfatin-1 also plays a principal role in other physiological activities such as adipose tissue differentiation, gastrointestinal motility, gluconeogenesis and insulin sensitivity, reproduction and, more importantly, cardiovascular function, as it is expressed in heart cells [2]. Recent data from animal models have revealed the actions of nesfatin-1 on cardiac modulation and blood pressure through various signaling pathways which affect the mechanical properties of the cardiac muscle, inflammation and oxidative stress, sympathetic activation and nitric oxide (NO) production, among others. On the other hand, human studies have shown a consistent association of nesfatin-1 levels with high-sensitive C-reactive protein (hsCRP), and low-grade inflammatory conditions like atherosclerosis, insulin resistance and, even more interestingly, CAD severity [3]. Dai et al. [4] were the first to observe a significant reduction in nesfatin-1 levels in patients with myocardial infarction, and, during the last five years, a small number of studies has put the association between nesfatin-1 levels and CAD under the spotlight [5–8].

Dipeptidyl peptidase 4 (DPP4) was initially recognized as a serine exopeptidase which cleaved amino-terminal dipeptides from the N-terminus of polypeptides in the extracellular space [9]. Its major role was considered to be the inactivation of the glucagon-like peptide-1 (GLP-1) and the glucose-dependent insulinotropic peptide (GIP), both members of the incretin hormone family, which leads to disrupted glucose metabolism and impaired insulin sensitivity. Recently, it has been demonstrated that DPP4 is expressed in a number of different tissues including the kidney, the liver, the adipose tissue and the heart, while it is also found in a soluble form in plasma, which derives from the shedding from the membranes of endothelial cells and exerts catalytic activity by cleaving oxyntomodulin and stromal cell-derived factor 1 (SDF-1) [10, 11]. Increased soluble levels and activity of DPP4 have been associated with increased glycosylated hemoglobin (HbA1c) and diabetes, dyslipidemia and chronic low-grade inflammation in both experimental and clinical situtations [12].Apart from its role in glucose homeostasis, DPP4 participates in functions such as smooth muscle cell proliferation, inflammation, apoptosis, and atherosclerosis regulation. Moreover, some studies have implied a possible role of DPP4 in the pathogenesis of heart failure and left ventricular dysfunction [13]. However, despite the overall negative association between DPP4 and coronary artery disease (CAD), several discrepancies have also been pointed out.

In this study, we aimed to investigate the association between nesfatin-1 and DPP4 levels and CAD prevalence and severity, along with the correlations of these enzymes with indexes of glucose regulation, lipid metabolism and chronic inflammation.

## Methods

### Study groups

This is a cross-sectional, observational study including participants with and without known significant CAD (227 men and 99 women with age range: 52–70 years). Diagnosis of significant CAD was established on either recent coronary angiography, defined as organic stenosis of at least ≥ 50% of the luminal diameter in one at least major coronary artery or history of coronary revascularization. Our participants were not receiving any DDP4 inhibitor and were divided into the following groups:

1. Unstable angina (UA, n = 112): Consecutive patients admitted to the coronary care units of our Cardiology Departments with unstable angina defined as recent onset of cardiac-origin symptoms on exertion or angina at rest and normal levels of cardiac troponin. All UA patients received standard anti-anginal and anti-platelet therapies, during hospitalization, according to international guidelines. Those who underwent coronary angiography and had significant CAD were recruited in our study.
2. Stable CAD (SCAD, n = 214): It was defined as asymptomatic patients with previous (within the last 3 years), angiographically documented, significant CAD, or history of either percutaneous coronary intervention or coronary artery bypass graft, with or without past myocardial infarction. Patients with SCAD and recent deterioration of symptoms were not eligible.
3. Control group (CG, n = 102): Age- and sex-matched to previous two groups participants were recruited from our cardiology outpatient clinics. All of them had at least two cardiovascular risk factors (diabetes, dyslipidemia, hypertension, obesity, positive family history for early CAD). In recent coronary angiography (within the last 3 years) their coronary arteries appeared normal or with mild stenosis (< 50%), while no recent history of acute coronary syndrome was present.

After initial selection, we further excluded patients using the following criteria: young age (< 40 years-old); history of recent, within the past 6 months severe chronic heart failure (class NYHA II–IV); the presence of severe concomitant pathologies such as cancer, recent major trauma/surgery, renal or liver insufficiency (creatinine > 2 mg/dl or ALT > 2 times upper normal limit, respectively), acute or chronic infectious disease, or any kind of auto-immune disease. Those exclusion criteria were deemed necessary in order to create more homogeneous groups, limiting the confounders of adipokines levels (e.g. pro-inflammatory conditions).

This study was conducted according to the Declaration of Helsinki and was approved by the local ethics committees of all participating hospitals. Written informed consent was obtained from each patient.

### Clinical and laboratory measurements

All participants underwent a complete medical history and a comprehensive physical examination. All co-morbidities, pharmaceutical regimens and smoking habits were recorded using a structured questionnaire. Blood pressure, body mass index (BMI), and ankle-brachial index (ABI) were obtained at baseline. Notably, we used blood pressure measurements away from the admission day (after 24 h) for UA patients. Diabetes, dyslipidemia and hypertension definitions have been previously set. Echocardiographic examination (Vivid E95; General Electric, Ohio, OH, USA) was performed to all participants by the same operator for the evaluation of left ventricular morphology and function.

Βlood samples were obtained just prior to the coronary angiography procedure and were separated and aliquoted for storage at − 80 °C until assayed. After overnight fasting, serum glucose, total cholesterol, triglycerides, low-density lipoprotein cholesterol (LDL-C) and high-density lipoprotein cholesterol (HDL-C) were determined enzymatically, using a chemical analyzer (Olympus, Japan). High-performance liquid chromatography (Menarini Diagnostics, Florence, Italy) was used for serum glycated hemoglobin A1c (HbA1c) determination. An IRMA kit was used to measure insulin level (DIAsource ImmunoAssays S.A., Louvain-la-Neuve, Belgium).

The inter- and intra-assay CV were 6.3 and 2%, respectively. Homeostatic model assessment of insulin resistance (HOMA-IR) was calculated from fasting insulin and fasting plasma glucose (FPG) by the following equation: HOMA-IR = fasting insulin (µU/ml) × FPG (mg/dl)/405. We measured high-sensitivity CRP (hsCRP) using a particle enhanced immunoturbidimetric assay (Hitachi 917 analyzer: Boehringer Mannheim, Germany). The detection limit was 0.1 mg/l, with intra- and inter-assay CVs of 1.34 and 5.7%, respectively. Serum concentrations of nesfatin-1 and DPP-4 were determined by an enzyme-linked immunosorbent assay (ELISA) kit.

### Coronary angiography

All participants underwent coronary angiography, and the severity of coronary artery stenosis was graded using the Gensini score. The latter was calculated from the number of stenotic coronary artery segments, the degree of their lumen stenosis and the localization of the stenosis (e.g., left main, proximal, mid, distal segments).

### Statistical analysis

Data were expressed as the mean with standard deviation (SD) for continuous variables, and frequencies for categorical variables. For continuous variables, the differences between two groups were examined by an independent samples t-test. More than two groups were compared by one-way ANOVA, using least significant difference as a post-hoc Tuckey test to compare individual groups. Skewed data were analyzed using the Mann–Whitney U test and Kruskal–Wallis H test. For categorical clinical variables, differences between groups were evaluated by chi-square tests. Pearson correlation coefficients were calculated to show the associations between nesfatin-1 or DPP-4 with the related variables. We explored the independent determinants of CAD in the whole study group using standard multiple regression analysis after controlling for other potential covariates. Similarly, we analyzed the Gensini score independent determinants within CAD population (UA & SCAD groups). A 2-sided p < 0.05 was considered significant. For the whole analysis we used SPSS 25.0 software (IBM, USA).

## Results

### Groups comparison

After initial evaluation and exclusion criteria application we finally considered eligible 76 patients for UA group, 165 patients for SCAD group and 85 patients for control group. Baseline characteristics of all participants are summarized in Table 1: no differences were found for age, sex hypertension and diabetes distribution between groups. The SCAD group had the lowest percentage of active smokers and the highest prescription rate of statins compared to the other two groups. The latter may explain the most favorable lipid profile observed in that group.

UA group showed significantly higher hsCRP than controls (p < 0.001) and SCAD (p = 0.011). HOMA-IR levels significantly differed between UA and control group (p < 0.001). Besides this, low nesfatin-1 levels were found in both CAD groups (UA & SCAD) with respect to controls (p < 0.001 & p = 0.002, respectively). The difference between UA and SCAD groups was marginally non-significant (p = 0.055). Regarding DPP4 we observed a significant reduction along UA to SCAD and control groups (p < 0.05) (Table 1). Although the presence of diabetes was associated with elevated DPP4 concentrations among all participants, the aforementioned significant differences between groups remained unchanged in non-diabetic participants (UA: 72.22 ± 15.13 ng/dl, SCAD: 56.92 ± 11.21, Control:35.92 ± 9.21; p < 0.05).

**Table 1:** Clinical, biochemical and angiographic characteristics of all study groups

	UA group (n = 76)	SCAD group (n = 165)	Control group (n = 85)
Males/females	55/21	112/53	60/25
Age (year)	66 ± 12	68 ± 12	64 ± 12
Smoking, n	52 (68.4%)	16 (9.8%)	30 (35.3%)*^#^
Hypertension, n	59 (77.6%)	131 (79.4%)	48 (56.5%)*
Diabetes, n	21 (27.6%)	42 (25.5%)	15 (17.6%)
Statins, n	45 (59%)	159 (96.4%)	29 (34.1%)*^#^
BMI (kg/m^2^)	30.42 ± 5.92	29.58 ± 3.71	29.4 ± 3.42
SBP (mmHg)	138 ± 24	132 ± 18	128 ± 15^#^
ABI	1.12 ± 0.31	0.98 ± 0.26	1.17 ± 0.39
DBP (mmHg)	81 ± 15	81 ± 11	77 ± 14
LVEF (%)	59 ± 10	61 ± 8	62 ± 7
TChol (mg/dl)	205 ± 36	165 ± 36	212 ± 48*
HDL-C (mg/dl)	39 ± 10	44 ± 17	47 ± 13^#^
LDL-C (mg/dl)	134 ± 61	95 ± 31	140 ± 40*
TG (mg/dl)	158 ± 92	128 ± 65	127 ± 73
FPG (mg/dl)	140 ± 57	131 ± 40	125 ± 32
HbA1c (%)^a^	7.6 ± 1	7.2 ± 1.1	7.1 ± 0.8
Insulin (mU/l)	12.2 ± 5.90	11.35 ± 5.02	9.01 ± 4.73
HOMA-IR	4.22 ± 1.91	3.67 ± 1.25	2.78 ± 1.18^#^
hsCRP (mg/l)	5.98 ± 2.562	3.59 ± 1.16	2.12 ± 0.88^#^
Nesfatin-1 (pg/ml)	331.75 ± 101.22	416.51 ± 119.83	515.12 ± 111.85^#^*
DPP4 (ng/dl)	81.92 ± 20.21	65.35 ± 14.67	42.81 ± 10.66^#^*

*n* number of patients, *BMI* body-mass index, *SBP* systolic blood pressure, *DBP* diastolic blood pressure, *ABI* ankle-brachial index, *LVEF* left ventricular ejection fraction, *TChol* total cholesterol, *TG* triglycerides, *FPG* fasting plasma glucose, *HOMA-IR* homeostasis model assessment-insulin resistance, *hsCRP* high-sensitivity CRP, *DPP4* Dipeptidyl peptidase 4

^#^UA vs. control group p < 0.05

*SCAD vs. control group p < 0.05

^a^HbA1c was measured only in the diabetic subgroup

### Correlations of nesfatin-1 and DPP4

We further searched for correlations of nesfatin-1 and DPP4 with the rest of variables, in the whole study group. Nesfatin-1 significantly negatively correlated with hsCRP (r = − 0.287, p = 0.036), HOMA-IR (r = − 0.587, p = 0.007) and hyperlipidemia (r = − 0.331, p = 0.034). In standard multiple regression analysis, hsCRP remained independent determinant of nesfatin-1 levels in the whole study population (R^2^ = 0.185, p = 0.024). On the other hand, DPP4 was significantly positively associated with hs-CRP (r = 0.353 p < 0.001) and FPG (r = 0.202, p = 0.020) in univariate analysis, but those correlations were lost in multiple regression analysis. In parallel, the presence of CAD related to age (r = 0.298, p = 0.004), hyperlipidemia (r = 0.243, p = 0.047), hsCRP (r = 0.559, p < 0.001), hypertension (r = 0.422, p < 0.001) and nesfatin-1 (r = 0.331, p = 0.034). After standard multiple regression age, nesfatin-1 and hsCRP appeared as independent determinant of CAD presence (Table 2).

**Table 2:** Logistic regression analysis with the presence of coronary artery disease as the dependent variable in the whole study group

	Ex(B)	95% CI	p value
Age	3.12	1.05–6.77	0.012
Hypertension	1.77	1.10–2.22	0.578
Hyperlipidemia	2.18	1.22–34.01	0.024
hsCRP	1.55	1.43–7.59	0.019
Nesfatin-1	2.74	0.41–6.91	0.016

*CI* confidence interval, *hsCRP* high-sensitivity C-Reactive Protein

We further proceeded to the evaluation of its relationship with CAD severity. For this purpose, we confined the analysis in the CAD groups, and we observed decreasing concentrations of nesfatin-1 across the number of diseased vessels (data not shown). Similarly, we observed a negative correlation between nesfatin-1 and the severity of CAD, quantified by the Gensini score (r = − 0.511, p < 0.001). Standard multiple regression revealed diabetes presence, hsCRP and nesfatin-1 to be independent determinants of the Gensini index in our CAD cohort (R^2^ = 0.177, p = 0.038) (Table 3). As expected, DPP4 levels did not vary significantly across the number of narrowed coronary arteries or the Gensini score (p > 0.05).

**Table 3:** Standard multiple regression analysis of Gensini score (dependent variable) and other independent variables within the CAD groups (UA & SCAD)

	Gensini score		
	β	95% CI	p value
Nesfatin-1	− 0.305	0.128–0.497	0.012
Diabetes	0.178	0.023–0.312	0.033
HOMA-IR	0.235	0.104–0.896	0.297
hsCRP	0.345	0.166–0.704	0.002

*CI* confidence interva, *hsCRP* high-sensitivity C-Reactive Protein, *HOMA-IR* homeostasis model assessment

## Discussion

In this study, serum nesfatin-1 levels were significantly lower, while serum DPP-4 levels were significantly higher in CAD groups (UA & SCAD) than control. We also observed a negative independent association of nesfatin-1 with the prevalence and severity of CAD, based on the number of diseased vessels and Gensini score. hsCRP seems to mediate the role of nesfatin-1 and DPP-4.

Our study revealed a statistically significant inverse association between nesfatin-1 and both stable and unstable CAD compared to controls, with also a remarkable, albeit marginally insignificant, difference between UA and SCAD. The effect of nesfatin-1 on cardiovascular performance has been demonstrated in many animal models, through pathways which regulate both cardiac function and blood pressure [3]. Angelone et al. [14] showed for the first time that nesfatin-1 induces a dose-dependent reduction of contractility and relaxation in isolated rat heart preparations through mechanisms such as cyclic guanosine monophosphate (cGMP)/protein kinase G (PKG) pathway or the natriuretic peptide receptor A (NPR-A), which led to reduced infarct size; in goldfish, a positive inotropic effect was associated with increased stroke volume and stroke work [15]. Intracerebroventricular (ICV) injection of nesfatin-1 increased blood pressure in rats via changes in the autonomic tone [16]; similarly, high peripheral levels of nesfatin-1 increased blood pressure in rats, probably through stimulating phosphoinositide-3-kinase (PI3K)/AKT/m-TOR pathway and phosphorylation of Janus kinase 2 (JAK2)/STAT3, which led to functional and structural changes of smooth muscle cells [17]. In humans, data about the cardioprotective effects of nesfatin-1 has generally been consistent and in line with our study. Kuyumcu et al. [5] recruited 67 NSTEMI patients and 33 healthy controls and evaluated the association between nesfatin-1 and the severity of CAD according to SYNTAX score, a scoring system which assesses the complexity of CAD based on lesion characteristics and is related to clinical outcomes, similarly to the Gensini score. Patients with MI had higher SYNTAX scores and nesfatin-1 levels, and a negative correlation between these two markers was shown. The same inverse association between nesfatin-1 and presence of CAD was shown by Dai et al. [4], who studied fasting plasma levels of nesfatin-1 in patients with acute myocardial infarction (AMI), or stable angina pectoris (SAP) and 34 control subjects. As we also showed, there was a negative correlation between nesfatin-1 and Gensini score, where patients with acute MI showed lower levels compared to stable angina pectoris and controls. Another two different cohorts showed a robust inverse association between its levels and the incidence of the no-reflow phenomenon after PCI treatment, an index of aggravated endothelial dysfunction and complexity of CAD [6, 7]. Notably, one study has doubted the cardioprotective effects of nesfatin-1, among patients undergoing elective coronary angiography, with CAD being present in 57% of them. CAD patients had higher nesfatin-1 levels compared to controls, (median 0.21 vs. 0.17 ng/mL, p < 0.01), and a stepwise increase was found across the increasing number of > 50% stenotic coronary vessels. More importantly, it was implied that nesfatin-1 levels were an independent risk factor for the presence of CAD (OR:1.71) [3]. It was postulated that these results could be attributed to the pro-inflammatory properties of nesfatin-1 which have been shown in some studies in patients with inflammatory diseases such as COPD [18] and osteoarthritis [19], along with its pro-hypertensive action. However, it must be noted that this study did not include any patients with acute coronary syndrome, which was a crucial methodological difference compared to ours and the aforementioned studies.

Apart from the traditional risk factors for atherosclerosis such as dyslipidemia and insulin resistance, inflammation and oxidative stress also play a pivotal role in every stage, including the development and the rupture of the atherosclerotic plaque [20]. Although, as we mentioned, nesfatin-1 can have both a pro-inflammatory role in some chronic inflammatory diseases, most research data favor its anti-inflammatory effects. In patients with head trauma, the administration of nesfatin-1 inhibited the activation of the nuclear factor kappa-B (NFκB) pathway and reduced the expression of inflammatory cytokines such as interleukin-6 (IL-6), IL-1 and tumor necrosis factor alpha (TNF-α) in rats [21]. In another murine model, intravenous nesfatin-1 alleviated gastric ulcers and reduced TNF-α and IL-1β by suppressing cyclooxygenase 2 signaling [22]. In our study, a significant negative correlation between nesfatin-1 levels and hsCRP was revealed, in accordance with previous data. Kuyumcu et al. [5] and Dai et al. [4] showed a similar correlation in their cohorts (r = − 0.535, p < 0.001 and r = − 0.330, p = 0.018, respectively); on the contrary, Ibe et al. [8] found no association between these two variables, but the absence of patients with acute CAD might have again been a confounding factor.

The negative univariate association between nesfatin-1 and HOMA-IR revealed in our study has not been a consistent finding. Dai et al. [4] and Ibe et al. [8] have failed to show any association between nesfatin-1 and HOMA-IR or HbA1c, while Sahin et al. [23], in a cohort of 54 patients with polycystic ovary syndrome (PCOS) and 48 matched controls, showed a strong positive correlation with HOMA-IR. A recent meta-analysis showed no statistically significant differences in circulating nesfatin-1 levels between patients with T2DM and controls (MD = − 0.04; 95% CI = − 0.32 to − 0.23), however, higher levels were noted in patients with newly diagnosed disease and lower levels in those under antidiabetic treatment [24]. The discrepancies in research data can be attributed to differences in study design and the complex neuronal and biochemical on which nesfatin-1 acts regarding glucose homeostasis; in general, the association between nesfatin-1 and diabetes is still vague. Finally, our study is the first to show a negative correlation between nesfatin-1 and lipids, as previous data have failed to show any association, even in CAD patients [4, 5]. A study in diabetic mice has shown that nesfatin-1 stimulates fatty-acid oxidation by activating AMP-activated protein kinase [25], while in another animal model, chronic subcutaneous infusion of nesfatin-1 reduced plasma cholesterol and triglyceride levels [26], however, further research in humans is needed to clarify the role of this adipokine in lipid metabolism.

Regarding DPP4, research data about its association with heart disease has been inconsistent. Our findings concur with the study by Yang et al. [27], which was the first to evaluate the possible association between DPP4 activity (DPP4a) and the presence of CAD. CAD patients has higher DPP4 levels (p < 0.01) and higher DPP4 activities (p < 0.01) compared to healthy patients without CAD, independently of the presence of diabetes mellitus (DM), and these results were even more marked in patients with acute MI or unstable angina compared to those with stable CAD. On the other hand, in the study by Li et al. [28] on percutaneous coronary intervention-treated (PCI) STEMI patients, a 1 U/L increase in DPP4a led to increased risk of major cardiovascular events (including MI) only in the subset of diabetic patients (HR:1.16). The reasons for the discrepancy with the previous results were assumed to be either the differences in the study cohorts, as the latter included only STEMI patients, or differences in the study design, as the cross-sectional nature which applies also to our study prevents the evaluation of long-term outcomes. Even more impressively, in another cohort including 747 PCI-treated STEMI patients [29], although higher DPP4a was associated with an increased rate of no-reflow events (OR:1.07), DPP4a was significantly lower in the STEMI patients compared to unstable angina (UA) controls (p < 0.0001), but it was not significantly different to that of the non-STEMI (NSTEMI) controls (p = 0.12). Similarly, in another ethnic group of 875 angiographically-proven CAD patients of European origin, a single-nucleotide polymorphism (SNP) in the DPP4 gene, which leads to decreased plasma DPP4 levels, was associated with increased risk of myocardial infarction [30]. More importantly, large randomized clinical trials (RCTs) on the cardiovascular outcomes of DPP4 inhibitors (DPP4is) have further failed to support any favorable cardiovascular effects of lower DPP4 levels, demonstrating only a noninferior risk of a composite CV outcome (CV death, nonfatal myocardial infarction, or nonfatal stroke) in diabetic patients with established cardiovascular disease [31].

The pathophysiological mechanisms behind these contradictory results have not yet been elucidated. DPP4 exerts its effects both in a catalytic and a non-catalytic pathway. The catalytic pathway involves its peptidase function by removing X-proline dipeptides from the N-terminus of polypeptides, and it is mainly responsible for its role in glucose homeostasis, while its non-catalytic functions involve its interaction with various ligands such as adenosine deaminase (ADA) and fibronectin, and it is mainly responsible for the stimulation of T cells and the cascade of chronic inflammation [13]. In experimental models, DPP4 inhibition has shown favorable effects on NO production, angiogenesis, blood pressure and myocardial function. DPP4^−/−^ mice showed improved survival after experimental infarction, and treatment with 250 mg/kg/d sitagliptin for 8 weeks led to improved functional recovery after ischemia/reperfusion (I/R) injury through increased endothelial cell density, reduced myocardial hypertrophy and enhanced fibrosis [32, 33]. Whether, however, these results could be directly attributed to direct GLP-1 actions on myocardial cells has been a matter of debate; recent data suggest a role for the GLP-1 dependent atrial natriuretic peptide, taking into account the fact that the myocardium lacks GLP-1 receptors [34]. In addition, it has been proposed that the soluble circulating form of DPP4 in plasma lacks the intracellular and the transmembrane regions of the membrane associated DPP4 [35]. As DPP4 acts not only on incretins but also on other substrates which are involved in cardiovascular disease, it is sensible to assume that the relative contributions of these substrates to the cardiovascular actions of DPP4 differ among patients or diseases, and it is thus the equilibrium between the catalytic and the non-catalytic function of DPP4 which ultimately determines its role, in both diabetic and non-diabetic individuals [13].

We showed a significant association of DPP4 with hsCRP and FPG in univariate analysis, although these correlations were lost after multiple regression analysis. Yang et al. also showed a similar association for CRP (r = 0.41, p < 0.01) which in contrast to our results, was associated with the presence and severity of CAD [27]. That study enrolled predominantly patients with diabetes and myocardial infarction which may explain the discrepancy of results. Nevertheless, the association between DPP4 and fasting plasma glucose has not been consistent. In the study by Li et al. [29], no association between these two parameters was found. In general, although a positive correlation between plasma DPP4 activity and FPG or HbA1c has been reported in some studies, no established causality has been proven [12]. In addition, it appears that the membrane-bound DPP4 form plays a more important role in the degradation of GLP-1 than the soluble form, so that it is quite doubtful whether the plasma DPP4 activity is a reliable index of the total DPP4 activity and, therefore, whether it affects glycemic control at all. High glucose concentrations promoted mRNA expression and membrane-bound DPP-4 activity in vitro [36]; on the other hand, intensified glycemic control, as expressed by a decrease in HbA1c of 1.5%, did not result in lower DPP4 activity in T2DM patients [37]. As in the case of myocardial function, the exact pathophysiological connections between DPP4 and glucose homeostasis are yet to be defined.

Our study has some limitations. These include the relatively small sample size and the fact that anthropometric measures which are important indexes of obesity and cardiometabolic disease, such as waist-to-hip ratio or waist circumference, were not obtained. In addition, the antidiabetic and anti-hypertensive medications were not documented and, therefore, it was not possible to investigate their impact on DPP4 and nesfatin-1 levels. Finally, we did not searched for their interaction with oxidative stress, regarding the very limited data in the current literature.

## Conclusions

This study revealed that serum DPP4 levels are higher in CAD patients, while serum nesfatin-1 levels have a negative association with both the incidence and the severity of CAD. These results are independent of the presence of diabetes mellitus. In addition, both peptides have a strong association with hsCRP, a fact which indicates an association with chronic inflammation, although more research in different cohorts needs to be conducted to clarify these correlations. These associations could present with an additive prognostic value while evaluating high cardiovascular risk individuals. Further large-scale studies are necessary to establish these associations and determine the role of DPP4 and nesfatin-1 in the pathogenesis of cardiovascular disease.

## Data Availability

The datasets used and/or analyzed during the current study are available from the corresponding author on reasonable request.

## Abbreviations

ABI: Ankle-brachial index
BMI: Body mass index
CAD: Coronary artery disease
CG: Control group
cGMP: Cyclic guanosine monophosphate
DBP: Diastolic blood pressure
DPP4: Dipeptidyl peptidase-4
FPG: Fasting blood glucose
GIP: Glucose-dependent insulinotropic peptide
GLP-1: Glucagon-like peptide-1
HbA1c: Glycosylated hemoglobin
HDL-C: High-density lipoprotein cholesterol
HOMA-IR: Homeostatic Model Assessment for Insulin Resistance
hsCRP: High-sensitive C-reactive protein
ICV: Intracerebroventricular
IL: Interleukin
LDL-C: Low-density lipoprotein cholesterol
LVEF: Left ventricular ejection fraction
NO: Nitric oxide
NPR-A: Natriuretic peptide receptor A
PCI: Percutaneous coronary intervention
PKG: Protein kinase G
SAP: Stable angina pectoris
SBP: Systolic blood pressure
SCAD: Stable coronary artery disease
SD: Standard deviation
SDF-1: Stromal cell-derived factor 1
STEMI: ST-elevation myocardial infarction
T2DM: Type 2 diabetes mellitus
T-CHOL: Total cholesterol
TG: Triglycerides
TNF-α: Tumor necrosis factor alpha
UA: Unstable angina
